# Integrated delivery of antiretroviral treatment and pre‐exposure prophylaxis to HIV‐1 serodiscordant couples in East Africa: a qualitative evaluation study in Uganda

**DOI:** 10.1002/jia2.25113

**Published:** 2018-05-31

**Authors:** Norma C Ware, Emily E Pisarski, Edith Nakku‐Joloba, Monique A Wyatt, Timothy R Muwonge, Bosco Turyameeba, Stephen B Asiimwe, Renee A Heffron, Jared M Baeten, Connie L Celum, Elly T Katabira

**Affiliations:** ^1^ Department of Medicine Brigham and Women's Hospital Boston MA USA; ^2^ Department of Global Health and Social Medicine Harvard Medical School Boston MA USA; ^3^ Department of Epidemiology and Biostatistics Makerere University College of Health Sciences Kampala Uganda; ^4^ STD Clinic/Ward 12 Mulago Hospital Kampala Uganda; ^5^ Harvard Global Cambridge MA USA; ^6^ Infectious Diseases Institute Makerere University Kampala Uganda; ^7^ Kabwohe Clinical Research Centre Kabwohe Uganda; ^8^ Department of Global Health University of Washington Seattle WA USA; ^9^ Department of Medicine Makerere University Kampala Uganda

**Keywords:** HIV prevention, evaluation, mechanism of effect, service delivery, pre‐exposure prophylaxis (PrEP), antiretroviral treatment (ART), implementation, Uganda

## Abstract

**Introduction:**

Serodiscordant couples are a priority population for delivery of new HIV prevention interventions in Africa. An integrated strategy of delivering time‐limited, oral pre‐exposure prophylaxis (PrEP) to uninfected partners in serodiscordant couples as a bridge to long‐term antiretroviral treatment (ART) for infected partners has been implemented in East Africa, nearly eliminating new infections. We conducted a qualitative evaluation of the integrated strategy in Uganda, to better understand its success.

**Methods:**

Data collection consisted of 274 in‐depth interviews with 93 participating couples, and 55 observations of clinical encounters between couples and healthcare providers. An inductive content analytic approach aimed at understanding and interpreting couples’ experiences of the integrated strategy was used to examine the data. Analysis sought to characterize: (1) key aspects of services provided; (2) what the services meant to recipients; and (3) how couples managed the integrated strategy. Themes were identified in each domain, and represented as descriptive categories. Categories were grouped inductively into more general propositions based on shared content. Propositions were linked and interpreted to explain “why the integrated strategy worked.”

**Results:**

Couples found “couples‐focused” services provided through the integrated strategy strengthened partnered relationships threatened by the discovery of serodiscordance. They saw in services hope for “getting help” to stay together, turned joint visits to clinic into opportunities for mutual support, and experienced counselling as bringing them closer together.

Couples adopted a “couples orientation” to the integrated strategy, considering the health of partners as they made decisions about initiating ART or accepting PrEP, and devising joint approaches to adherence. A couples orientation to services, grounded in strengthened partnerships, may have translated to greater success in using antiretrovirals to prevent HIV transmission.

**Conclusions:**

Various strategies for delivering antiretrovirals for HIV prevention are being evaluated. Understanding how and why these strategies work will improve evaluation processes and strengthen implementation platforms. We highlight the role of service organization in shaping couples’ experiences of and responses to ART and PrEP in the context of the integrated strategy. Organizing services to promote positive care experiences will strengthen delivery and contribute to positive outcomes as antiretrovirals for prevention are rolled out.

## 1 Introduction

HIV serodiscordant couples – in which one partner is HIV‐infected and the other uninfected – are a priority population for delivery of new HIV‐prevention interventions in Africa. Oral antiretroviral medications are effective in preventing HIV infection, both when taken as treatment (ART) 1, 2, and when taken as pre‐exposure prophylaxis (PrEP) by uninfected persons 3, 4, 5.

The Partners Demonstration Project (Clinicaltrials.gov NCT02775929) was a prospective implementation study evaluating an integrated strategy of delivering ART and PrEP to serodiscordant couples in African public health settings 6, 7. The integrated strategy offered time‐limited PrEP to uninfected partners as a “bridge” to long‐term ART in the infected partner. Uninfected partners were offered PrEP at baseline and encouraged to discontinue once infected partners had used ART for six months. One thousand and thirteen (N = 1013) heterosexual HIV‐1 serodiscordant couples at high risk for HIV infection according to a validated risk score 8 participated. The research took place at four sites in Kenya and Uganda. The evaluation investigated: (1) uptake of and adherence to PrEP and ART, (2) continuing use of PrEP following initiation, and (3) the integrated strategy's effectiveness in preventing HIV infection.

Results revealed the integrated strategy to be highly successful. Rates of uptake of PrEP and ART were 97% and 91% respectively. PrEP adherence was high, as measured by return rates of dispensed pills, electronic medication event monitoring (MEMS) and levels of drug detected in blood plasma 6, 7, 9. Ninety‐five‐percent (95%) of uninfected partners initiating PrEP at enrolment were still receiving it after three months. Only four incident HIV infections occurred across the study population during the two‐year follow‐up period, for an observed HIV‐1 incidence of 0.24 per 100 person‐years. This represents a reduction of 95% in the rate of expected new infections, compared to a counterfactual simulation in which expected HIV incidence was calculated to be 4.9 per 100 person‐years 6, 7.

Outcomes of the Partners Demonstration Project indicate the integrated strategy is an effective, cost‐effective, and implementable approach to delivering PrEP and ART to African serodiscordant couples to prevent HIV infection 6, 7, 10. Once a new model of healthcare delivery has been shown to work, it becomes important to understand *how* and *why* it works, to facilitate future replication and scale‐up.

We evaluate the integrated strategy as implemented at two Partners Demonstration Project sites. The evaluation is based on qualitative data. It examines the organization of services and couples’ responses to address the question: Why did the integrated strategy of delivering PrEP and ART to East African discordant couples ‘work’ to prevent transmission of HIV?

## 2 Methods

### 2.1 Research setting

The qualitative evaluation was carried out at the Project's two Ugandan sites: the Infectious Diseases Institute – Kasangati, outside Kampala; and the Kabwohe Clinical Research Center, in Kabwohe.

### 2.2 Sampling and recruitment

Purposeful sampling, in which participants are deliberately selected to construct a varied study sample, supports in‐depth investigation in qualitative research 11. Here, purposeful sampling was used to identify couples with varying experiences of PrEP and ART. We also included couples where the HIV‐uninfected partners declined, as well as accepted, PrEP, and couples in which HIV‐infected partners were both ineligible and eligible for ART at enrolment. (At study outset, only HIV‐infected individuals with CD4 counts ≤350 were eligible for treatment; Ugandan national guidelines were revised in 2016) 12. Demonstration Project participants falling into these categories were referred by Ugandan study staff. Research assistants approached these individuals during clinic follow‐up visits for the Demonstration Project to describe the qualitative research, answer questions, and invite participation. Ninety‐three (N = 93) couples took part.

### 2.3 Data collection

Two types of qualitative data collection activities were used. Multiple, in‐depth individual and joint interviews were conducted with couples at different time points, to represent variation in experiences of the integrated strategy. Initial interviews were joint interviews, to allow for exploration of relationship dynamics. Subsequent interviews were a combination of individual and joint sessions, depending on the topic or topics to be discussed. Field observations focusing on services provided to couples were also carried out.

#### 2.3.1 In‐depth Interviews

In‐depth interviews were conducted approximately one month after Partners Demonstration Project enrolment, at later points in the follow‐up period, and when key “transitions” in experiences of the integrated strategy occurred. Examples of events prompting “transition” interviews were: (1) ART initiation, (2) PrEP discontinuation, (3) separation from partner, and (4) missed clinic visits. Follow‐up interviews covered a variety of relevant topics, whereas transition interviews focused on examining a particular experience. Two hundred and seventy‐four (N = 274) interviews were completed: 93 initial interviews, 88 “follow‐up” interviews; and 93 “transition” interviews. One hundred and twenty‐six (N = 126) were individual interviews; 55 (44%) of individual interviewees were men; 71 (56%) were women.

Interviews were conducted by trained Ugandan research assistants in local languages (Luganda, Runyankore), using interview guides. Each interview type had a different guide, tailored to the experience being investigated. For example, baseline interview topics included: (1) discovery of serodiscordance, (2) decisions to accept or decline PrEP and ART, (3) early experiences of taking PrEP and ART, (4) understandings of PrEP, (5) impact of PrEP on the partnered relationship, (6) perceptions of the integrated strategy. A sampling of questions used in study interview guides appears in the Appendix.

Interviews were conducted in private settings in locations reflecting interviewee preferences. Participants gave written consent for interviews, which were audio‐recorded and lasted about an hour. Audio‐recordings were transcribed into English by the research assistants. Interview data were collected from November 2013 through December 2016.

#### 2.3.2 Field observations

Field observations are a well‐established data collection technique in qualitative research that involve the presence of a researcher in a naturalistic setting, the witnessing of events and activities of interest, and sometimes a degree of participation 13. Observations provide a direct view of phenomena under study and thus complement the mediated perspective obtained through interview data.

Here, observations focused on clinical encounters between couples and health care providers implementing the integrated strategy, including interactions with counsellors, physicians and pharmacists. Screening/enrolment visits, quarterly follow‐up visits, and study exit visits were observed on randomly selected dates with permission from all parties. Research assistants observed interactions and took notes, but did not actively participate. Fifty‐five observations were completed, lasting 2½ hours on average. Consent for observations was confirmed verbally before each observation session. Observers wrote up what they had seen as field notes – complete narrative descriptions of the interactions. Observational data were collected from March 2014 to March 2016.

### 2.4 Data quality

Translated transcripts and field notes were reviewed for content and technique in weekly feedback phone calls and emails. Feedback focused on ensuring texts were rendered in clear, standard English. Harvard team members conducted periodic in‐person visits to study sites for additional support and “refresher” trainings.

### 2.5 Data analysis

An inductive, content analytic approach 14 was used for data analysis. Interview transcripts and field notes were reviewed as they were produced, to provide a general sense of data content and emerging themes. A coding scheme was developed based on the initial review; data were coded using Atlas.ti software. Coded data and field notes were re‐reviewed to formulate themes in three domains: (1) key aspects of services provided, from couples’ perspectives; (2) what these services meant to couples; and (3) how couples managed the integrated strategy. Themes formed the basis for category development. Resulting categories were labelled using statements summarizing their meaning, elaborated to specify their content, and illustrated using excerpts from the data. Categories were grouped inductively based on shared content into more general propositions. Propositions were linked and interpreted to propose an explanation of “why the integrated strategy worked.”

### 2.6 Ethical approvals

Ethical approvals were obtained from the Committee on Human Studies, Harvard Medical School, Boston MA, USA; the National HIV/AIDS Research Committee of the Ugandan National Council for Science and Technology, Kampala, Uganda; and the University of Washington Institutional Review Board, University of Washington, Seattle, WA, USA.

## 3 Results

### 3.1 Participant characteristics

Partners Demonstration Project eligible couples were ≥18 years of age, sexually active, and reported intending to remain together. Characteristics of couples participating in the qualitative study appear in Table 1.

**Table 1:** Characteristics of couples participating in the qualitative evaluation study (N = 93 couples)

	Median (IQR) or N (%)
	Total
Characteristics, uninfected partner	
(Female) sex	43 (46%)
Age, years	31 (26 to 37)
Education, years	7 (5 to 10)
Monthly income, any	91 (98%)
Initiated PrEP at Project enrolment	82 (88%)
Initiated PrEP at enrolment or during Project follow‐up period	86 (92%)
PrEP Adherence, MEMS cap (MEMS cap bottle openings/expected openings) (N = 84)^a^	91% (66 to 98)
Characteristics, infected partner	
Age, years	31 (25 to 37)
Education, years	7 (6 to 10)
Monthly income, any	62 (66%)
ART eligible, Project enrolment	61 (66%)
CD4 cell count/μL	472 (293 to 708)
Initiated ART within 15 days of Project enrolment, eligible individuals (N = 60)	40 (67%)
Time to ART for eligible individuals not initiating within 15 days of Project enrolment, days (N = 20)	84 (33 to 168)
Initiated ART at some point during Project follow‐up period (N = 91)^b^	91 (100%)
Characteristics, couple	
Living together, years	3 (1 to 9)
Married to each other	91 (98%)
Children together	1 (0 to 2)
Time since learning about serodiscordance, months	2 (1 to 12)

a MEMS cap data are not available for 9 participants.

b ART initiation data are not available for 2 participants.

Infected and uninfected partners in the qualitative study were in their early thirties. Approximately half (46%) of uninfected partners were female. Eighty‐eight percent (N = 82) of uninfected partners initiated PrEP at enrolment; 66% (N = 61) of infected partners were eligible for ART at enrolment; 67% (N = 40) of eligible individuals initiated ART at enrolment. Median PrEP adherence, measured through electronic monitoring, was 91%.

### 3.2 Qualitative results

#### 3.2.1 Overview

We propose an explanation for the success of the integrated strategy in preventing HIV transmission in Ugandan serodiscordant couples taking part in the Partners Demonstration Project. The explanation draws together analytic insights in three areas: (a) how services were organized to implement the integrated strategy; (b) how couples experienced this organization; and (c) how couples managed the integrated strategy. Essentially, we propose: (1) services were organized to be “couples‐focused” in ways that couples experienced as strengthening their partnered relationships, and as a result, (2) couples adopted a “couples orientation” to the integrated strategy. In the following Results section, these key findings are summarized as “propositions,” and elaborated using descriptive categories (Subsections of 3.2.2 and 3.2.3). Data excerpts illustrating category content appear in Tables 2 and 3. A summary linking the propositions interpretively completes our proposed answer to the question of “why the integrated strategy worked.”

**Table 2:** Data excerpts illustrating proposition 1: couples‐focused services were experienced as strengthening serodiscordant relationships

Category Summary	Elaboration	Data excerpts
A. In services designed for serodiscordant couples, couples saw hope for staying together.	“Getting help” to stay together	“The idea of joining the project] “gave us courage because we expected to get help. When we reached [the Project clinic] we got counselling and got to know the world is like that [serodiscordance can happen]. They provided us with our first help.” *–Female, HIV‐uninfected partner, Age 32* “*…* it is not like we were joining the project to get jobs or other benefits. We saw that joining this study would help us and also others in the world, because the research results might help other people to live together as discordant couples.” *–Male, HIV‐infected partner, Age 48*
	Serodiscordance as a condition of enrolment	“…We work on serodiscordant couples only. If you are to continue with us, one of you has to be HIV positive and another one HIV negative*…*” *–Field Observation, May 22, 2014*
B. Attending follow‐up appointments as a couple brought partners together, increasing mutual support.	Travelling and waiting room time provided couples with time for discussion, reflection and joint decision making.	*“…*. We travelled together and talked together on the way there. I would say, ‘knowing what the clinic staff say, what do you really think?’ …I could ask her, ‘they told me this, but what should we do?’ We could advise each other.” *–Male, HIV‐uninfected partner, Age 24*
	Accompanying infected partners to clinic to ensure continued access to HIV care.	“I go to [clinic] to help her. … if I terminate my participation there, …they may not help her.” *–Male, HIV‐uninfected partner, Age 37* “*…* you put up a clinic …for discordant. Can you get medicine from there if you went as an individual? I think you have to go as a couple. [If] my husband goes to the clinic alone, they may … ask him ‘where is your wife?’ So I have to continue going to [name of clinic] to support my husband.” *–Female, HIV‐uninfected partner, Age 21*
C. Partners experienced counselling for HIV prevention as bringing them closer to each other.	Counselling messages characterized HIV as a shared experience to be managed by partners together.	A pharmacist urges an uninfected partner to take responsibility for reminding his wife to take her medication, as she initiates ART. “The pharmacist advised the participant to remind his wife to take the drugs. The participant replied, ‘But she told the counsellor that she will take them!’ The pharmacist added, ‘Yes, she can say that and then she forgets. It's normal. You remind her, since she is just starting.*’*” *–Field observation, June 25, 2014*
	Appreciation of guidance on managing the crisis of serodiscordance to avoid separation.	Woman: “… since we started coming here, we have been getting counselling. When we go back [home] we put what we are taught… into practice. If it was not for counselling, we wouldn't be together. When the counsellor teaches you, you settle down.” Interviewer: *[To male partner]: “*What can you add, sir?*”* Man: *“*Yes, change is there. …. If it had not been for counselling, I think I wouldn't be having my wife up to this time. When we were tested and found out that we are discordant, I thought my wife was going to leave me, since she was negative. But because of counselling, we are together and she is very supportive up to now. She welcomes me when I come home, she cooks food and we eat and she is not discriminating and hating me because of my status.” *– Male HIV‐infected partner, Age 28; Female HIV‐uninfected partner, Age 21*
	Guidance on how to behave as a couple was gratefully received and had a positive impact.	“We were handed to one lady, who welcomed us, then started asking us questions about what we go through as a couple.…We talked to her about our problems…and she counselled us, telling us how we were to conduct ourselves.[She taught us] how to cooperate as a couple because between us there was quarrelling….[She] said, ‘now each of you has to give the other respect. You have to love each other and should not say: why am I positive and you are negative?’…Going to [Demonstration Project clinic site] helped me so much because my home is now peaceful. We are one, we love each other, and we cooperate, compared to back before we went there. Those people have done a good job in our lives.” *–Female, HIV‐uninfected partner, Age 44*
D. Simultaneous use of ARVs turned management of HIV into a shared experience.	Experience of taking antiretrovirals together helped couples “settle” … back into their relationship.	Man*: “*I think it is better for a negative partner to be given PrEP at the same time a positive partner is taking ART …It has helped us; it introduced peace at home. … Because we both take medicine and we take it at the same time, we remind ourselves, sit together and take [the pills] and each one asks the other how he or she feels. It has continued to make us settle. Woman*:* By him seeing me taking PrEP without quarrelling, like ‘you brought HIV and you are making me take medicine,’ that made him settled and also to love me more. This was because I did not quarrel or treat him in a bad way. I could bring water and sometimes open the tin, remove medicine and give him to take whenever it was time to take medicine.” *– Female, HIV‐uninfected partner, Age 32, Male, HIV‐infected partner, Age 33*

**Table 3:** Data excerpts illustrating proposition 2: couples adopted a “couples‐orientation” to the integrated strategy

Category summary	Elaboration	Data excerpts
E. Concern for partner wellbeing was a reason for initiating ART.	Desire to protect one's partner as a reason for initiating ART	*“*I told her, ‘I am going to use ARVs purposely for you because I want you to be protected.’ That is what I told her. I was not to take it for myself. I was not going to start ARVs if it was for me.” *–Male, HIV‐infected partner, Age 30* M: “…I told them that I want to be the first security towards my wife.*” I: “*What do mean by security?” *M: “*Although she is swallowing [PrEP], it is I who has to be her first security. … I have to be her security and know how I handle her so that she does not get the virus.” *–Male, HIV‐infected partner, Age 40*
F. Reinforcing the partnered relationship was a reason to accept PrEP.	Resolving the serodiscordance crisis as a reason for accepting PrEP	*“…* we started loving each other when we were still young. We promised each other love until death, so when I suggested separation after I found out that we are discordant, she did not want to breach our agreement. … She told me ‘Let me take PrEP to protect myself from HIV other than separating from you because I still love you.*’”* **–** *Male, HIV‐infected partner, Age 24*
G. Couples devised joint strategies for adhering to PrEP and ART.	Mutual reminders	*“…* when I am with her in the house and the time to swallow her medicine arrives, I remind her. She also reminds me when time arrives to swallow my medicine, because we help each other and we agree with each other.” *–Male, HIV‐uninfected partner, Age 36*
	Emotional and material support for adherence	*“*He takes ART well and … I also take good care of him. I do not worry him, I give him food in time, I make sure I give him drinks like passion juice, fruits and that helps him.” *–Female, HIV‐uninfected partner, Age 32*

#### 3.2.2 Proposition 1: Couples‐focused services were experienced as strengthening serodiscordant relationships

##### In services designed for serodiscordant couples, couples saw hope for staying together

Testing for HIV and learning that one partner was HIV negative and the other HIV positive came as a shock to most couples, who until that moment may not have known serodiscordance was possible. Typically, discovery of serodiscordance created a crisis for partnered relationships. Neither partner wished to abandon the relationship, but no alternative to separation seemed available.

Couples struggling with the crisis of serodiscordance saw in the Partners Demonstration Project hope for avoiding separation. Knowing little before their first visit, they understood only that the Project represented an opportunity for people “like them.” Serodiscordance was poorly understood in local communities, and the use of antiretrovirals for prevention was unknown. Couples were initially drawn to the idea of participation as a way of “getting help” to stay together (Table 2, A).

The Project was perceived in local communities as a service for serodiscordant couples, as well as a research study. That couples, and not individuals, were to be the “unit of service” was consistently communicated. During introductory visits with couples who might participate, project staff made it clear that serodiscordance was a condition of enrolment, and made the meaning of the term explicit (Table 2, A). Word spread through the community that new services designed specifically for serodiscordant couples were available.

##### Attending follow‐up appointments as a couple brought partners together, increasing mutual support

Couples were asked to attend follow‐up appointments jointly. During these visits, couples sat together in consultations with clinicians, counsellors, and pharmacists. In this way, receiving healthcare became a shared experience: partners heard the same information at the same time, and asked and had their questions answered as a couple.

Joint visits represented for couples an opportunity to spend time together. Visits afforded time to talk, a luxury not routinely available during busy days filled with work and child care. Travelling to and from clinic, and waiting together between consultations, couples had time to discuss what they were hearing, reflect, and make decisions together (Table 2, B).

Being together at clinic visits was an occasion for mutual support. Infected persons interpreted their partner's presence at clinic visits as a sign of continued interest and willingness to invest in the relationship. Uninfected partners saw themselves as supporting their infected spouses during visits by ‘distracting’ them with conversation. Some uninfected partners – believing the visit was more for the “sick” partner than for them – felt they were showing support simply by being there. These individuals saw themselves as accompanying their partners to clinic to ensure continued access to HIV care (Table 2, B).

##### Partners experienced counselling for HIV prevention as bringing them closer to each other

Counselling for participating couples took place at enrolment and follow‐up visits. Through standardized messages developed for the Project, counselling provided education on serodiscordance, use of PrEP and ART for prevention, and the integrated strategy of delivery 15. Counsellors presented PrEP as an HIV prevention tool for use during periods of greatest HIV risk, rather than as a life‐long intervention 15. Messages were communicated across the various components of follow‐up visits, that is, not only in interactions with Project counsellors, but also in consultations with physicians and pharmacists.

The serodiscordant relationship was treated as a resource for HIV prevention in developing counselling messages 15. In practice, we see how particular messages that at one level target HIV prevention, at another level may function to bring partners closer together. Explanations that serodiscordance is real, but need not signal the end of a relationship eliminated the need for separation to prevent HIV transmission. Cautions against sexual relations with outside partners encouraged partners to turn toward each other. Recommending that partners take responsibility for reminding each other to practice HIV prevention – through regular condom use, and daily medication adherence – characterized HIV infection as a shared experience, to be managed by partners together (Table 2, C).

Couples appreciated and came to rely on the counselling they received during follow‐up visits. They experienced project staff as caring individuals who treated them with respect, listened to their concerns, and continuously provided encouragement and support. High praise was offered, in particular, for what couples experienced as guidance on managing the crisis of serodiscordance to avoid separation (Table 2, C).

Interviewees also credited other forms of counselling support with strengthening, even saving, their relationships. Reassurance that with treatment, HIV could be managed as a chronic illness, alleviated concerns about early death and disability, calming fears and ending quarrels. Encouraging shared responsibility for dosing with PrEP and ART turned adherence into a joint enterprise. Finally, counsellors’ clear and specific instructions on how to behave as a couple – what to do to get along – were gratefully received and had a positive impact (Table 2, C).

##### Simultaneous use of ARVs turned management of HIV into a shared experience

The integrated strategy meant both partners used antiretrovirals simultaneously for several months. Taking antiretrovirals during the same time period created a sense of HIV as a shared experience – a situation partners handled together. Over time, the experience of taking antiretrovirals together helped couples move past the shock and upset that accompanied discovery of serodiscordance, and “settle,” as they put it, back into their relationship (Table 2, D).

#### 3.2.3 Proposition 2: Couples adopted a “couples orientation” to the integrated strategy

The integrated strategy required that infected partners initiate ART, uninfected partners “accept” PrEP, and both adhere to the medication. Couples could be seen prioritizing and drawing upon their relationship as they negotiated these expectations.

##### Concern for partner wellbeing was a reason for initiating ART

Ugandan national guidelines defining ART eligibility became more inclusive during the Partners Demonstration Project. This meant many infected persons had the option of beginning treatment before experiencing symptoms. At the same time, participating couples were also learning about the use of ART for prevention.

Accordingly, infected persons’ rationales for beginning treatment broadened beyond a focus on preserving personal health, to include a concern for partner wellbeing. When asked why they were initiating ART, interviewees not infrequently cited a desire to please and/or protect their partner (Table 3, E).

##### Reinforcing the partnered relationship was a reason to accept PrEP

A desire to reinforce the partnered relationship shaped HIV‐uninfected partners’ decisions to accept PrEP. Some saw PrEP use as a way of expressing solidarity with partners taking ART. Others embraced PrEP as a means of remaining uninfected while staying in the serodiscordant relationship. Resolving the serodiscordance crisis was a powerful motive for taking a chance with this unknown medication, in that it meant preserving emotional ties and honouring long‐standing commitments (Table 3, F).

##### Couples devised joint strategies for adhering to PrEP and ART

Couples understood the importance of good adherence, and they tried hard to take PrEP and ART as recommended – at the same time, every day. Typically, they approached adherence jointly – sharing responsibility for making sure each took his/her medication correctly. Shared responsibility for adherence took various forms. Three of these were: (a) dosing together, (b) mutual reminders and (c) emotional and material support.

Couples who dosed together took their pills in each other's presence. For these couples, dosing had a ritual quality. They would come together, set out the pills, bring water to take them with, and sit face‐to‐face while taking the medication, sometimes drinking from the same glass. Some couples experienced dosing together as bringing them closer as a couple.

Couples who did not dose together nevertheless made a point of knowing each other's dosing schedules and following each other's dosing behaviours. If one partner had not seen the other taking the medication at dosing time, s/he was likely to offer a reminder. Reminders were communicated in person, and via phone calls and/or texts (Table 3, G).

Partners also made efforts to provide each other with emotional and material support for adherence. They encouraged each other to continue taking pills daily, and worked to create a nurturing environment that would make the task of daily pill‐taking easier. Male partners provided money to ensure medications could be taken with food. Women prepared food, worked to create a stable daily routine, and tried to limit stress for partners taking ART (Table 3, G).

#### 3.2.4 Linking propositions to explain “Why the integrated strategy worked”

We offer two propositions, drawn from qualitative data, to explain ‘why the integrated strategy worked’. The first is that couples experienced the integrated strategy's “couples‐focused” approach to service organization as strengthening serodiscordant relationships. The second is that couples tended to adopt a “couples orientation” to the integrated strategy.

**Figure 1:**
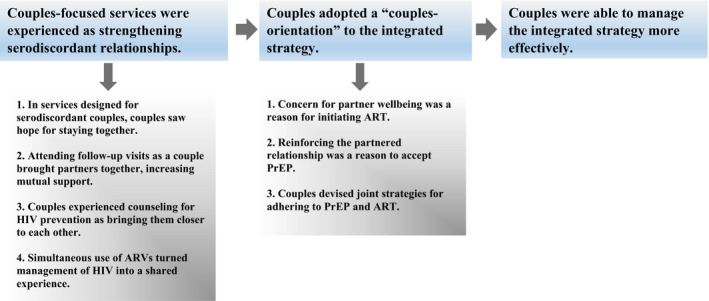
A proposed explanation of why integrated delivery of PrEP and ART to East African serodiscordant couples “worked” to prevent HIV transmission.

We propose that this couples orientation arose, at least in part, from the new solidarity couples derived from couples‐focused services. Their management of the integrated strategy may have been more effective as a result. Better management of the integrated strategy may have translated to a favourable impact on ART initiation, PrEP acceptance, and PrEP and ART adherence – lowering rates of HIV transmission, and helping to make the integrated strategy a success (Figure 1).

## 4 Discussion

The process of making PrEP and “immediate” ART available outside research settings is gaining momentum in Africa. Informing this process is currently a priority for research. The integrated strategy of delivering PrEP and ART to serodiscordant couples has been shown to be implementable and effective. We have unpacked the implementation process in Uganda to posit a mechanism of effect.

Much qualitative research on antiretrovirals for prevention in Africa reports user perspectives – attitudes, knowledge, preferences, and influences on the use of PrEP and immediate ART 16, 17, 18, 19, 20, 21, 22, 23, 24, 25, 26. Qualitative methods have also been used to shed light on reasons underlying suboptimal PrEP adherence in the VOICE and FEM‐PrEP clinical trials 27, 28, 29, 30, 31, 32.

This study draws on user perspectives to look for insights into outcomes of the Partners Demonstration Project. We focus on service delivery in the integrated strategy, and on how participating couples responded. Other PrEP demonstration projects, as well as combination prevention and rapid treatment studies, employ diverse strategies for delivering interventions 33, 34, 35, 36, 37. This qualitative evaluation study is among the first to examine how these strategies are put into practice and what they mean to those involved, thereby helping to explain their mechanisms of effect 38.

Results of this study overlap with a conceptual model of health behaviour change proposed by Lewis et al. (2006) 39. The model lays out a mechanism explaining how being part of a couple may shape individual health behaviours. The logic is that a couple's interdependence leads partners to perceive threats to health as impacting the relationship. This perception prompts a “communal coping” response in which couples work together to reduce health threats. We have seen how HIV infection is experienced as weakening partnered relationships for serodiscordant couples in this and other studies 40. We have also seen partners thinking in relational terms when deciding to accept PrEP (“to stay together”) and initiate ART. “Communal coping” is reflected in the joint approach couples may adopt to adherence. In these ways, our findings corroborate Lewis et al.'s “interdependence and communal coping” model of health behaviour.

Serodiscordant couples participating in this study experienced integrated strategy services as strengthening their relationship. Nevertheless, not all couples remained together. Twenty‐one (23%) couples in the qualitative study reported ending their relationship after enrolling in the Partners Demonstration Project. Many of these couples struggled with additional challenges that may or may not have stemmed from discovery of serodiscordance. Among them were perceived infidelities, refusal to practice HIV prevention, and failure to provide material support.

Results of this qualitative evaluation amplify results of the Partners Demonstration Project itself. Several limitations should be recognized. First, our proposed explanation is intended as a *partial* explanation. We acknowledge the contributions of factors other than service organization (and medication efficacy) to the integrated strategy's success. Second, as qualitative research, this study does not claim generalizability of results. Results should, however, be *transferable* in ways that inform future implementation of the integrated strategy in African public health settings. Third, the proposed explanation should be understood as suggestive, rather than definitive. Framing the explanation as propositions deliberately sets the stage for critical examination through additional research. Fourth, our analysis is based on data from the Partners Demonstration Project's Ugandan sites only. Any differences in how the integrated strategy was implemented in Kenya are not captured here. Finally, member‐checking was not part of this qualitative research design.

## 5 Conclusions

Various strategies for delivering antiretrovirals for HIV prevention are being evaluated – in Africa and elsewhere. An understanding not only of whether, but how and why these strategies work (or do not work) should be part of the evaluation process, to create the strongest possible implementation platforms. Thorough understanding requires a broad focus that acknowledges the complexity of implementation processes and incorporates multiple intervention components.

We highlight the role of service organization as a component of effective strategies for delivering antiretrovirals for HIV prevention. In the integrated strategy, services organized to be “couples‐focused” strengthened relationships threatened by serodiscordance. We propose strengthened partnered relationships improved couples’ responses to and use of PrEP and ART by fostering a “couples orientation”. Organizing services to promote positive care experiences will strengthen delivery and contribute to positive outcomes as antiretrovirals for prevention are rolled out.

## Competing interests

The authors have no competing interests to declare.

## Authors’ contributions

NCW and MAW designed the qualitative research. NCW led the data analytic process and wrote and revised the manuscript. MAW and EEP provided general supervision for the data collection process in Uganda, contributed to the data analytic process and reviewed and commented on early drafts of the manuscript. TRM, ENJ, ETK, SBA and BT supervised data collection at the Ugandan sites. JMB, CLC and RAH provided feedback on emerging findings from the qualitative study and reviewed and provided comments on a preliminary draft of the manuscript. All authors critically reviewed and approved the current version of the manuscript.

## Appendix 1

### 1.1

**Table:**

Interview type	Sample questions from study interview guides
Initial interview: Joint	How did you find out about being serodiscordant?
	How do you feel about serodiscordance?
	After you were given your [screening test] results, what did you tell the staff you decided to do about taking PrEP (and ART, if eligible)? How did you make this decision?
	In this study, HIV‐uninfected partners are offered PrEP to reduce the risk of HIV infection when the (HIV‐infected) partner is [waiting to start ART (for ineligible)] or [has just started ART (for ART eligible)]. What is your opinion of this?
	What do you think the possibility of [HIV‐uninfected partner] acquiring HIV now that you both are in the Demonstration Project? Why? What about before you joined the study?
Transition interview: PrEP discontinuation	How do you feel about stopping PrEP?
	How will you protect yourself from acquiring HIV? What changes will you make now that you've stopped PrEP, if any?
	If there was an opportunity for you to resume taking PrEP, would you? Why?
	When was the most recent time you missed taking a dose of your PrEP? What happened?
Transition interview: separation	What led you and your partner to separate?
	How has your separation from your partner changed your participation in the Demonstration Project?
	Since separating with your study partner, what changes have there been in the way you take your medicine?
Final interview	While [participating in this project], you and your study partner were offered ART and PrEP as part of an HIV prevention strategy to prevent the uninfected partner from acquiring HIV. How do you feel this prevention strategy has worked for you and your partner?
	Tell me how it was when your partner was taking ARVs and you were taking PrEP at the same time.
	Tell me about your last Demonstration Project follow‐up visit. Who did you see? What did you discuss?
	You continued attending Kasangati clinic for approximately two years. What were some of the reasons you continued to go to the clinic after you stopped taking PrEP?
